# The role of community development in improving awareness, diagnosis and management of childhood non-communicable diseases (NCDs) in Indonesia

**DOI:** 10.1186/1687-9856-2015-S1-P83

**Published:** 2015-04-28

**Authors:** Aman Pulungan, I Wayan Bikin Suryawan, Kate Armstrong, Dwi Lestari Pramesti

**Affiliations:** 1 University of Indonesia, Jakarta, Indonesia; 2 Cipto Mangunkusumo Hospital, Jakarta, Indonesia; 3 Indonesia Pediatric Society, Jakarta, Indonesia; 4 Caring and Living as Neighbours (CLAN), Australia

**Keywords:** children, NCDs, community development, support group, access to medicines

## Background

NCDs are now recognised as a major public health problem globally. In Indonesia, WHO estimates that 63% of all deaths are caused by NCDs. Lack of community awareness and health systems ill-equipped to deal with NCDs contribute to misdiagnosis, underdiagnosis, and increased preventable morbidity and mortality, particularly for children and adolescents.

## Objective

To raise awareness and improve the diagnosis and management of childhood NCDs in Indonesia.

## Method

Established NCD Family Communities: IKADAR (Type-1 Diabetes) in 2003, YTI (Turner’s Syndrome) in 2003, KAHAKI (Congenital Adrenal Hyperplasia) in 2008 and FOSTEO (Osteogenesis Imperfecta/OI) in 2013. Community development approach, with focus on education, research (collation of data from IPS registries at Cipto Mangunkusumo Hospital), advocacy and health systems strengthening.

## Results

Establishment of clubs and registers increased known prevalence across Indonesia: a 4.5 fold increase in CAH, from 65 patients (2008) to 293 (2014); 2 fold increase in OI, from 35 patients (2013) to 70 (2014); 5 fold increase in Diabetes, from 156 patients (2009) to 960 (2014). Access to medicines improved: donations of hydrocortisone (oral and injectable) and fludrocortisone co-ordinated by KAHAKI and CLAN, with efforts to register nationally; bisphosphonates included in national insurance scheme following launch of FOSTEO. Translation of educational resources and focused training for health professionals coincided with reduced presentations of children to hospital in adrenal crisis and diabetic ketoacidosis and reduced mortality from CAH and Diabetes. Media reports increased across all groups and information sharing amongst community members enhanced by WhatsApp.

## Conclusions

The experiences of NCD Communities in Indonesia offer insights into practical steps that can be taken to redress the inequitable plight of young people living with NCDs in the Asia-Pacific region. A community development focus drives sustainable change,and helps increase awareness. Awareness makes a difference.

